# Concentration dependent cholesteryl-ester and wax-ester structural relationships and meibomian gland dysfunction

**DOI:** 10.1016/j.bbrep.2020.100732

**Published:** 2020-01-30

**Authors:** Zofia A. Hetman, Douglas Borchman

**Affiliations:** Department of Ophthalmology and Visual Sciences, University of Louisville, Louisville, KY, 40202, USA

**Keywords:** Cholesteryl ester, Dry eye, Infrared spectroscopy, Meibum, Wax ester, CB, cholesteryl behenate, CE, cholesteryl ester, FTIR, Fourier transform infrared, OO, oleyl oleate, TFLL, tear film lipid layer, ν̃_sym_, the frequency of the infrared symmetric stretching band, WE, wax ester

## Abstract

**Background:**

With dry eye, the ratio of cholesteryl ester (CE) to wax ester (WE) decreases substantially in meibum, but the functional and structural consequences of this change are speculative. The aim of this study is to confirm this finding and to bridge this gap in knowledge by investigating the effect of varying CE/WE ratios on lipid structure and thermodynamics.

**Methods:**

Infrared spectroscopy was use to quantify CE and WE in human meibum and to measure hydrocarbon chain conformation and thermodynamics in a cholesteryl behenate, stearyl stearate model system.

**Results:**

The CE/WE molar ratio was 36% lower for meibum from donors with dry eye due to meibomian gland dysfunction compared with meibum from donors without dry eye. CE (5 mol %) dramatically increased the phase transition temperature of pure WE from -0.12 °C to 63 °C in the mixture. Above 5 mol % CB, the phase transition temperature increased linearly, from 68.5 °C to 85 °C. In the ordered state, CE caused an increase in lipid order from about 72% *trans* rotamers to about 86% *trans* rotamers. Above 10% CE, the hydrocarbon chains were arranged in a monoclinic geometry.

**Conclusions:**

The CE/WE is lower in meibum from donors with dry eye due to meibomian-gland dysfunction. Major conformational changes in the hydrocarbon chains of wax and cholesteryl ester mixtures begin to occur with just 5% CB and above.

**General significance:**

CE-WE interactions may be important for in understanding lipid layer structure and functional relationships on the surface of tears, skin and plants.

## Highlights

• The CE/WE is lower in meibum from donors with meibomian-gland dysfunction.
• CE may be important for the tear film lipid layer structure and function.
• CE-WE interactions may be important on the surface of tears, skin and plants.
• Conformational changes in WE and CE mixtures begin to occur with just 5% CE.
• CE, cholesteryl ester; WE, wax ester.

## 1 Introduction

**Fig. 1:**
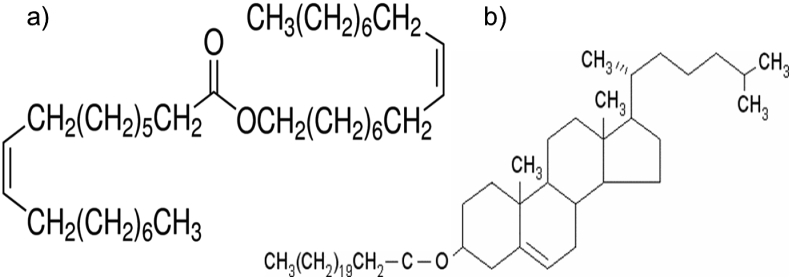
Structure of a) the wax ester oleyl oleate and b) the cholesteryl ester cholesteryl behenate.

Elucidating wax ester (WE) and cholesteryl ester (CE) compositional, structural and functional relationships is key to our understanding of how these lipids are involved in natural and pathological processes. WE and CE are neutral lipids (Fig. 1) found in abundance in: sebum, produced by sebaceous glands in the skin [1,2]; meibum [3,4], produced by the meibomian glands in the eyelids; the spermaceti of whales [5], the exoskeleton coating of insects [6] and the cuticle of plants [7,8]. Meibum CE have unusually long hydrocarbon chains [3,9] and are not miscible in phospholipid membranes [10,11].

The function of CE in many systems has been studied. In humans, CE regulates the cellular levels of free cholesterol [12] and transports cholesterol to specific organs [13]. Regulation of cholesterol by CE in in the ocular lens could be important as cholesterol levels are extremely high in the human lens, and ocular cholesterol levels are related to lifespan and cataract formation [[14], [15], [16]]. CE became widely studied when it was discovered that the concentration of CE increases to 50% with age in human aortic intima [17] and may contribute to plaque formation that plays a role in atherosclerosis [18,19]. Patients with sensitive scalp, a dermatological condition recently linked to abnormal amounts of sebum and have higher amounts of CE in their skin lipid than those without the condition [20].

The functions of WE are diverse. Epicuticular wax protects plants from ultraviolet radiation, pathogens and transpiration [6]. WE on the exoskeleton of some desert insects prevents water loss and serves to reflect sun light [21]. In humans, ‘ear wax’ protects the eardrum from debris [22]. In many marine species such as some whales, WE provides buoyancy, is a storage depot for lipids and plays a role in the reception and transmission of sound [5].

With dry eye, one of the most prevalent ophthalmologic complaints encountered by ophthalmologists [23,24], the ratio of CE to WE decreases substantially in meibum, but the functional and structural consequences of this change are speculative [4,25]. CE and WE constitute a majority of the human tear film lipid [[25], [26], [27], [28], [29], [30]]. Upon blinking, lipid from the meibomian glands in the eyelid spreads onto the ocular surface [[31], [32], [33]] and it is thought to influence tear stability [26,[34], [35], [36], [37]]. Relevant to the current study, the structural relationships between CE and WE could be important to understanding the function of tear lipids and dry eye disease as well as dermatological conditions. Synthetic CE at 50 mol % increased the phase transition temperature [38] of synthetic WE, but the relationship between the amount of CE and structural changes is unknown. The aim of this study is to bridge this gap in knowledge by investigating the effect of varying CE/WE ratios on lipid structure and thermodynamics using Fourier transform infrared spectroscopy (FTIR) as in previous studies [39,40]. Knowing the concentration-structural relationships could lead to a better understanding of the natural roles of CE and WE and their contribution to diseases, especially those related to meibum and sebum abnormalities where the percentage of CE changes.

## 2 Materials and methods

### 2.1 Materials

Oleyl oleate (OO) was obtained from Sigma-Aldrich Corp. (St. Louis, MO, USA), cholesteryl behenate (CB) from Santa Cruz Biotech (Dallas, TX, USA), and CHCl_3_ from Fisher Chemical (Pittsburgh, PA, USA). Potassium chloride crystal optic discs were obtained from Alfa Aesar (Haverhill, MA, USA).

### 2.2 Sample preparation

CE - WE mixtures: 0, 5, 10, 34, 40, 53, 65 and 100 mol % CE were made from stock solutions of CB and OO in CHCl_3_ (20 mM). Mixtures and stock solutions were stored at -70 °C under argon to prevent oxidation.

### 2.3 Fourier transform infrared spectroscopy, CE/WE standards

FTIR samples were prepared by placing 100 μL of each sample (0 to 100 mol % CB) onto a 13 × 2 mm, KCl infrared sample window. These were placed in a vacuum chamber for 5 min to evaporate off any remaining CHCl_3_. The KCl windows were placed within a temperature controlled infrared cell holder containing an insulated water coil connected to a Neslab RTE-111 (NESLAB Instruments, Newton, NH) circulating water bath. A thermistor touching the cell window enabled measurement and control of the sample cell temperature. The water bath unit was programmed to measure the temperature of the thermistor and to adjust the bath temperature so that the desired sample cell temperature was reached. Temperatures were maintained within ±0.01 °C.

Infrared spectra were obtained using a Thermo Nicolet Nexus 670 FTIR instrument (Thermo Fischer Scientific, Waltham, MA). They were acquired using 50 scans and a resolution of 1.0 cm^-1^. Analysis of the data was carried out using GRAMS/386 software (Galactic Industries Salem, NH).

The frequency of the infrared CH_2_ symmetric stretching band near 2850 cm^-1^, ν̃_sym_, was used to estimate the content of *trans* and *gauche* rotamers in the hydrocarbon chains as described previously [39,40]. In order to calculate the frequency of the symmetric stretch, ν̃_sym_, the baseline of the OH–CH stretching region near 3500 and 2700 cm^-1^ was leveled. Next, the center of mass of the CH_2_ symmetric stretching band was calculated by integrating the top 10% of the intensity of the band. The baseline for integrating the top 10% of the intensity of the band was parallel to the OH–CH region baseline. Lipid CH_2_ groups can present as either *trans* rotamers, which appear in ordered hydrocarbon chains, or *gauche* rotamers, prevalent in disordered hydrocarbon chains. Therefore, lipid hydrocarbon chain order can be evaluated based on the amount of CH_2_ *trans* rotamers present. As such, ν̃_sym_, which is dependent on the respective amount of *gauche* and *trans* rotamers [39,40], can be used to characterize lipid phase transitions [[39], [40], [41], [42], [43], [44], [45], [46], [47], [48], [49]] and to measure the *trans* rotamer content of lipid hydrocarbon chains with changes in temperature [39,[41], [42], [43], [44]]. Since rotamers are either *trans* or *gauche*, phase transitions can be described by a 2-state sigmoidal equation [40]. The percentage of *trans* rotamer data was used to calculate the phase transition enthalpy and entropy from the slopes of Arrhenius plots [40]. Arrhenius plots from tear-lipid phase transitions were linear with correlation coefficients greater than 0.998.

### 2.4 Fourier transform infrared spectroscopy, CE/WE molar ratio from human meibum

A standard curve from the infrared spectra of 34, 40, 53, 65 and 100% CB mixed with OO was used to determine the mole % of cholesteryl ester from infrared spectra of meibum. The area of the vibrational modes of the cholesterol sterol ring [50] near 800 cm^-1^ divided by the area of the CH_2_ twisting modes [51] of the hydrocarbon chains near 720 cm^-1^ (*area ratio*) was plotted on the Y axis. The mole % CB mixed with OO was plotted on the X axis. Infrared spectra from the CB/OO mixtures of standards described above were used to construct a standard curve to be used to measure the relative amount of CE/WE in human meibum samples. The *area ratios* of the standards were averaged from 11 infrared spectra for each concentration measured above 20 °C. Infrared spectra of human meibum were analyzed retrospectively from infrared spectra used to measure meibum lipid phase transitions [52]. Written informed consent was obtained from all donors. Protocols and procedures were reviewed by the University of Louisville Institutional Review Board as well as the Robley Rex Veterans Affairs Institutional Review Board. All procedures were in accord with the Declaration of Helsinki.

### 2.5 Statistics

Data are represented as the average ± the standard error of the mean. Significance was determined using the Student's *t* test. Values of *P* < 0.01 were considered statistically significant.

## 3 Results

Infrared spectra were analyzed retrospectively from infrared spectra used to measure meibum lipid phase transitions [52]. Patient demographics are presented in Table 1. The cohort of controls without dry eye was younger with less Blacks and males and more Asians compared with the cohort with meibomian gland dysfunction (MGD).

**Table 1:** Patient demographics.

Parameter	Control No Dry Eye	Meibomian Gland Dysfunction
Average Age ± Standard Deviation (y)	35 ± 17	66 ± 13
Median Age (y)	27	68
Race (%)	Caucasian (77.8)	Caucasian (77.8)
	Hispanic (3.7)	Hispanic (2.8)
	Black (3.7)	Black (11.1)
	Asian (14.8)	Unknown (8.3)
Gender (%)	Male (59.3)	Male (72.2)
		Unknown (2)
Number	27	36

**Fig. 2:**
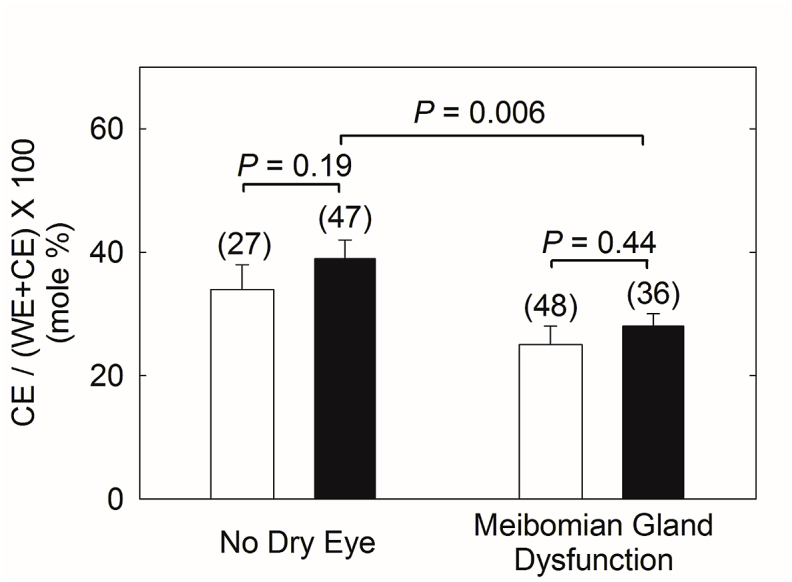
Cholesteryl ester content of human meibum calculated from infrared spectra (see Methods and Results) from the current study (open bars) compared with that calculated using NMR spectroscopy reported previously (filled bars) [4].

The % CE for meibum samples (Fig. 2) was interpolated from a standard curve from CB/OO mixtures (see Methods). The standard curve was linear with a correlation coefficient of 0.9987, an intercept of 0.087 *area ratio* and a slope of 2.37 *area ratio/*% CB. The average relative standard deviation of the *area ratios* for a given value % of CB was ±13% of the average value. The CE/WE molar ratio was 36% lower for meibum from donors with dry eye due to meibomian gland dysfunction compared with meibum from donors without dry eye (Fig. 2). There was no correlation between age and the CE/WE molar ratio for meibum from donors with dry eye due to MGD compared with meibum from donors without dry eye, r = 0.013 and 0.13, respectively.

**Fig. 3:**
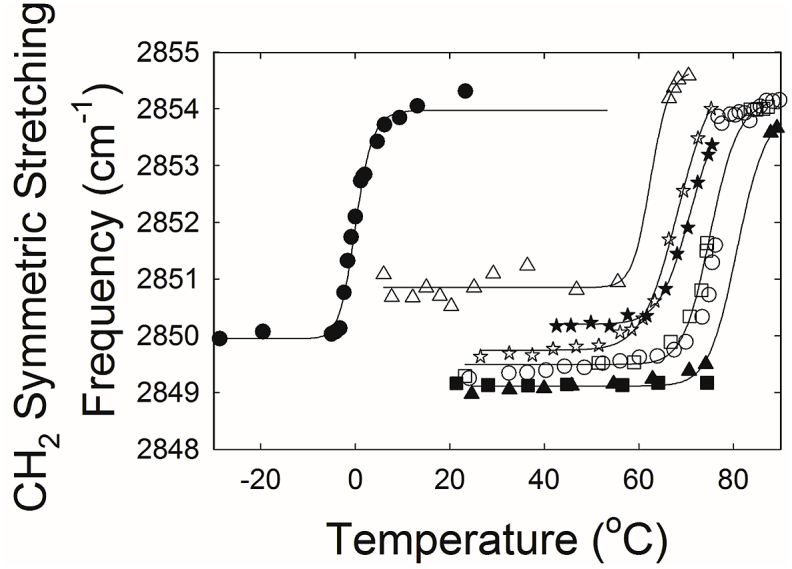
Phase transitions of cholesteryl behenate (CB) and oleyl oleate mixtures. The CH_2_ symmetric stretching frequency was used to measure lipid order (stiffness). The higher the frequency the more disordered are the hydrocarbon chains. (●) 0% CB, (Δ) 5% CB, (★) 10% CB, (✰) 34% CB, (O) 40% CB, (□) 53% CB, (▲) 65% CB and (■)100% CB.

**Fig. 4:**
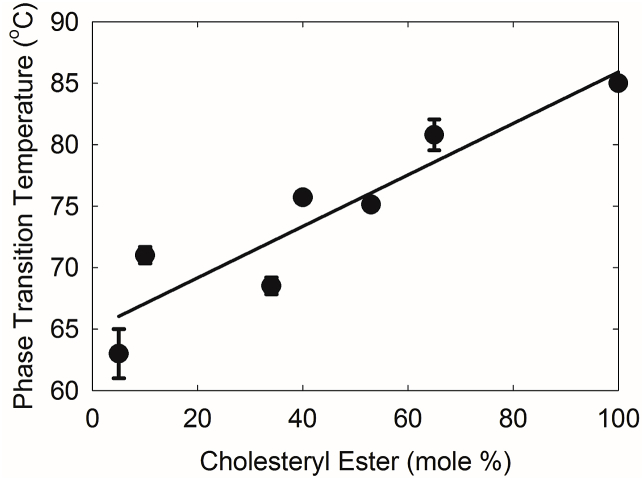
The change in the phase transition temperature with the mole % cholesteryl behenate mixed with oleyl oleate. The line is the linear regression fit to the data. The phase transition temperature is the temperature at the midpoint of the phase transition from an ordered gel phase to a disordered liquid crystalline phase.

Phase transitions may be used to measure lipid-lipid thermodynamic interactions. As expected, ν̃_sym_ increased with increasing temperature indicating a phase transition from an ordered, gel phase to a disordered liquid crystalline phase (Fig. 3). CB at a concentration of as low as 5 mol % dramatically increased the phase transition temperature of pure OO from -0.12 °C to 63 °C in the mixture (Fig. 4, Table 2, *P* < 0.001). Above 5 mol % CB, the phase transition temperature increased linearly and significantly (P < 0.01), from 68.5 °C to 85 °C (Fig. 4 and Table 2), but relatively smaller than the change from 0 to 5% CB.

**Table 2:** Phase transition data for mixtures of cholesteryl behenate (CB) in oleyl oleate (OO).

Phase Transition Parameter (CB mol %)	0	5	10	34	40	53	65
Minimum Wavenumber (cm^-1^)	2850.0 ± 0.1	2850.2 ± 0.5	2850.20 ± 0.02	2849.74 ± 0.04	2849.56 ± 0.09	2849.49 ± 0.08	2849.11 ± 0.04
Maximum Wavenumber (cm^-1^)	2854.0 ± 0.1	2854.7 ± 0.3	2854.37 ± 0.3	2854.98 ± 0.3	2854.03 ± 0.10	2854.2 ± 0.1	2854.03 ± 0.28
Phase Transition Temperature (°C)	-0.1 ± 0.2	63 ± 2	71.0 ± 0.7	68.5 ± 0.7	75.7 ± 0.1	75.1 ± 0.4	81 ± 1
Cooperativity (relative)	-17 ± 2	-32 ± 15	-20 ± 2	-17 ± 2	-98 ± 17	-26 ± 4	-26 ± 4
Order at 25.8 °C (% *trans*)	24.8 ± 0.4	69.43 ± 0.01	70 ± 2	76.4 ± 0.6	79 ± 1	80 ± 1	84.9 ± 0.6
Temperature at 50% order (°C)	-0.6 ± 0.2	61 ± 2	69.2 ± 0.6	66.6 ± 0.6	75.6 ± 0.1	74.8 ± 0.4	81 ± 1

**Fig. 5:**
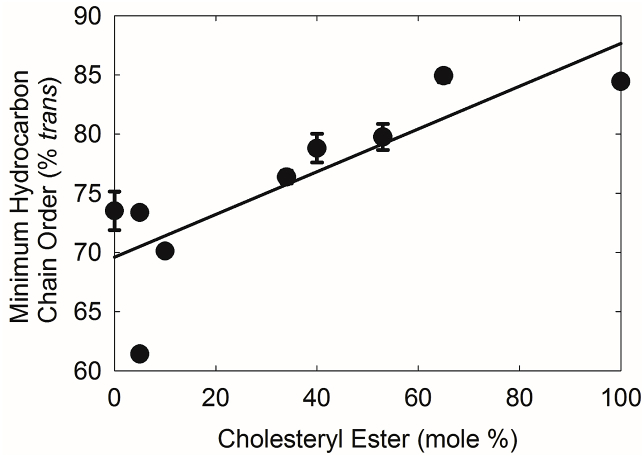
With increasing amounts of CE, the minimum ν̃_sym_ decreased indicating that in the ordered state, increasing CE concentration caused an increase in lipid order (% *trans* rotamers) from about 72% *trans* rotamers to about 86% *trans* rotamers. The line is the linear regression fit to the data.

With increasing amounts of CB, the minimum ν̃_sym_ decreased (Table 2) indicating that in the ordered state, increasing CB concentration caused an increase in lipid order (% *trans* rotamers) from about 72% *trans* rotamers to about 86% *trans* rotamers (Fig. 5, r = 0.9165, *P* < 0.01). There was no statistical difference (*P* > 0.05) between the Δ enthalpy and Δ entropy of all of our samples which averaged 378 ± 20 kcal/mol and 1.1 ± 0.1 kcal/mol/^o^C, respectively.

**Fig. 6:**
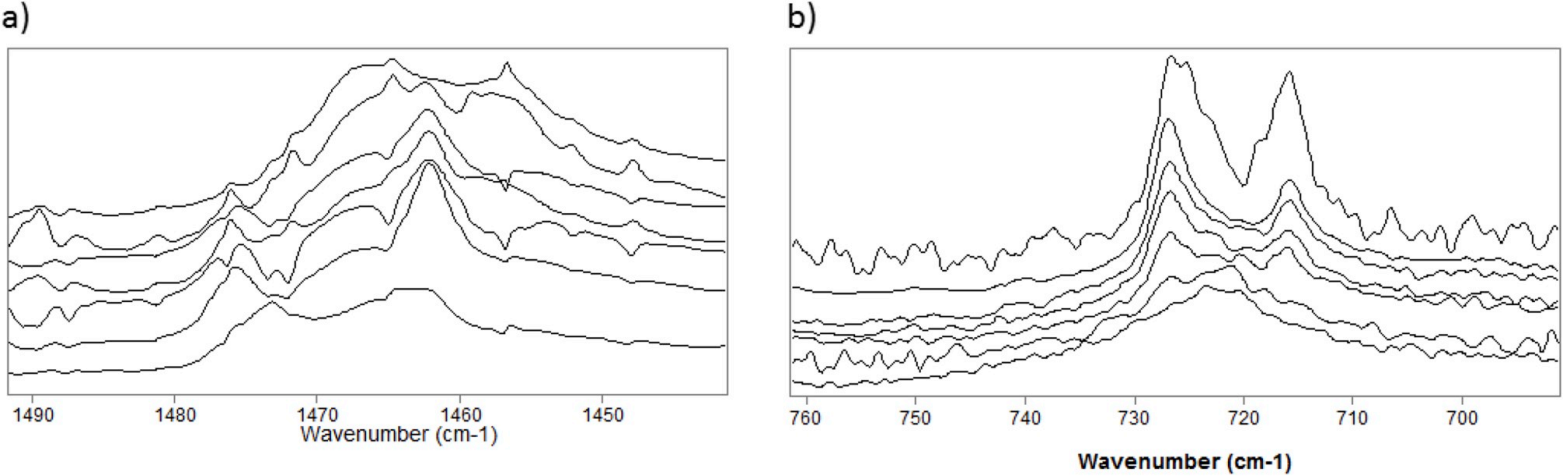
a) Infrared spectra of CH_2_ bending region for different concentrations of cholesteryl behenate (CB) mixed with oleyl oleate at room temperature. From top to bottom (mole %): 0% CB at 23.33C; 10 %CB at 24.63C; 34% CB at 26.37C; 40% CB at 24.01C; 53% CB at 23.00C; 65% CB at 24.05C; 100% CB at 21.27C. b) Infrared spectra of the CH_2_ rocking region of CB mixed with oleyl oleate at room temperature. From bottom to top (mole %): 0% CB at 23.33C; 10 %CB at 24.63C; 34% CB at 26.37C; 40% CB at 24.01C; 53% CB at 23.00C; 65% CB at 24.05C; 100% CB at 21.27C.

The band(s) between 1450 and 1480 cm^-1^ (Fig. 6a) were assigned to C–H in-plane bending of the hydrocarbon groups. While only a single peak is visible in the spectra of 0% and 10% CB, a second band near 1477 cm^-1^ appears in the spectra above 10% CB. The band(s) between 700 and 740 cm^-1^ were assigned to methylene rocking modes (Fig. 6b). For 0% and 10% CB mixtures, only one peak is discernible. Above 10% CB, peak splitting becomes apparent, with the peaks becoming increasingly distinct with greater amounts of CB. The splitting of the CH_2_ bending band and rocking bands is due to correlation field splitting and suggest that the hydrocarbon chains are arranged in an orthrombic or monoclinic geometry [40,53].

## 4 Discussion

There are two major findings of the current study: The 36% decrease in the meibum CE/WE ratio with MGD was confirmed and major packing and conformational changes in the hydrocarbon chains begin to occur with just 5% CB and above. As little as 5 mol % CB raised the phase transition temperature of the wax OO from -0.1 °C to 65 °C. In a relatively ordered wax ester mixture, CE at 50% increased the phase transition of WE by 14 °C [38]. The finding from the current study that only 5% CB can do so with a greater magnitude change is a new finding. Above 5 mol % CB, the phase transition temperature increased linearly with increasing CB, but the change was not as great as the change between 0 and 5% CB.

The idea that CE increases the lipid phase transition temperature and lipid hydrocarbon order, depending on the temperature, could be important to the function of the tear film lipid layer (TFLL). Upon blinking, tears are distributed across the surface of the cornea, which then ‘break up’ after about 10 s (tear breakup time) in normal individuals [54]. The TFLL is believed to aid the spreading of aqueous tears and contributes to their stability. The TFLL must be stiff enough to withstand the sheer force of a blink but fluid enough to spread across the aqueous tear layer [55]. The current study indicates that a small percentage, 5%, of CE would be expected contribute to the overall stiffness of the TFLL that enables it to withstand the sheer force of a blink.

Splitting of the CH_2_ bending modes near 1470 and CH_2_ rocking modes near 720 above 10% CE indicates a change in packing from a hexagonal to an orthorhombic or monoclinic geometry. The correlation between the splitting of CH_2_ bending and rocking bands and orthorhombic/monoclinic packing geometry has been made with phospholipids [60,61], triglycerides [62,63], monolaurin [64], and fatty acids [65]. The TFLL is thought to be multilayered and consist of a polar lipid region and a non-polar lipid region [66]. While the polar lipid layer seems to play a crucial role in stabilizing tears, CE was shown to change orientation under increase lateral pressure, potentially impacting tear film stability [67]. Tighter lipid packing or altered packing geometry could lead to less potential for reorientation within the non-polar lipid layer and therefore affect normal TFLL elasticity properties.

Although stiff ordered lipids are probably important for tear film stability, too much order may be detrimental, as it may keep meibum from flowing out of the meibomian glands. Meibum lipid order correlates with tear film stability with age between 0 and 25 years old [63], MGD [52,56], and with donors who have had hematopoietic stem cell transplantation [52,57]. Correlation does not necessitate cause, but the relationship between hydrocarbon chain order and tear film stability is intriguing. When tear film stability is restored with treatment, lipid order is also restored to normal levels [58] suggesting the relationship between lipid order and tear film stability may be more than coincidental. A more ordered lipid could contribute to the formation of a discontinuous patchy tear film lipid layer, which in turn results in deteriorated spreading [59], and decreased surface elasticity. One may speculate that more ordered lipid results in attenuated capability to restore tear film lipid layer structure between blinks. A detailed review on the topic has been published [37].

In the current study, the CE/WE molar ratio was shown to be 36% lower for meibum from donors with dry eye due to MGD compared with meibum from donors without dry eye, confirming a previous study in which lipid was quantified using nuclear magnetic resonance spectroscopy [4]. Concurrent with a decrease in the CE/WE ratio, lipid order increases with dry eye due to MGD [52,56]. The current study can address the question, is the decrease in lipid order with dry eye due to the observed decrease in the CE/WE ratio with dry eye? The current study showed that a decrease in very ordered CE from 46%, similar to the ratio measured for normal meibum, to 20%, similar to the molar percentage in meibum from donors with dry eye, would decrease lipid order of a fluid WE and ordered CE mixture from 80% *trans* to 74% *trans.* Thus, based on our data using synthetic lipids, a decrease in CE cannot be responsible for increased meibum order with dry eye due to MGD.

However, unlike the disordered synthetic WE used in the current study, human meibum WE would be expected to be very ordered due to long hydrocarbon chains and high saturation levels. Several species of CE and WE have been identified in meibum [3,9,28,30,[68], [69], [70], [71], [72]], and these could produce effects not accounted for in the current study. For ordered synthetic WE below the phase transition, CE linearly increased the order of the hydrocarbon chains, but had no effect on fluid WE above the phase transition temperature. Based on these observations, a decrease in CE could contribute to the increase in lipid order observed with dry eye [52,56,57].

In addition to the contribution of CE to lipid order discussed above, more CH_2_ moieties (fewer CH_3_ moieties) [73] could contribute to the higher order of meibum from donors with dry eye [52,56,57]. Fewer CH_3_ moieties may be due to less hydrocarbon chain branching [76]. As double bonds also contribute to lipid disorder [74,75], fewer double bonds could also contribute to a more ordered meibum. Experiments are currently underway to isolate CE and WE from human meibum so that they can be used for experiments under more physiological conditions.

## 5 Conclusions

The CE/WE is lower in meibum from donors with meibomian-gland dysfunction.

CE may be important for the tear film lipid layer structure and function. Major conformational changes in the hydrocarbon chains of wax and cholesteryl ester mixtures begin to occur with just 5% CB and above.

## CRediT authorship contribution statement

**Zofia A. Hetman:** Conceptualization, Data curation, Formal analysis, Investigation, Methodology, Validation, Writing - original draft. **Douglas Borchman:** Conceptualization, Data curation, Formal analysis, Funding acquisition, Investigation, Methodology, Project administration, Resources, Software, Supervision, Validation, Visualization, Writing - original draft, Writing - review & editing.
